# Bromido-1κ*Br*-tricarbonyl-2κ^3^ *C*-(2η^5^-cyclo­penta­dien­yl)molybdenum(I)tungsten(I)(*W*—*Mo*)

**DOI:** 10.1107/S1600536808012828

**Published:** 2008-05-07

**Authors:** Martin O. Onani, Jan-André Gertenbach, Muhammad D. Bala

**Affiliations:** a University of the Western Cape, Modderdam Road, Bellville, Cape Town 7535, South Africa; b Department of Chemistry and Polymer Science, University of Stellenbosch, Private Bag X1, Matieland 7602, South Africa; c School of Chemistry, University of KwaZulu-Natal, Westville Campus, Private Bag X54001, Durban 4000, South Africa

## Abstract

The title compound, [WMoBr(C_5_H_5_)(CO)_3_], is built up from a pseudo-square-pyramidal piano-stool coordination around the Mo atom, the important geometry being Mo—W = 2.6872 (7) Å, W—Br = 2.5591 (9) Å and Mo—W—Br = 158.35 (3)°.

## Related literature

For related literature, see Albright *et al.* (1978 ▶); Bueno & Churchill (1981 ▶); Changamu *et al.* (2006 ▶); Friedrich *et al.* (2004 ▶).
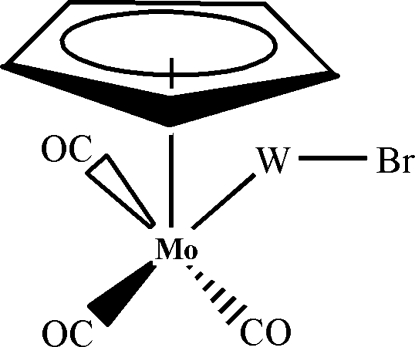


## Experimental

### 

#### Crystal data

[WMoBr(C_5_H_5_)(CO)_3_]
*M* *_r_* = 508.82
Tetragonal, 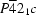

*a* = 11.9375 (9) Å
*c* = 15.546 (2) Å
*V* = 2215.4 (4) Å^3^
*Z* = 8
Mo *K*α radiation
μ = 15.09 mm^−1^
*T* = 100 (2) K
0.11 × 0.10 × 0.07 mm

#### Data collection

Bruker APEX CCD area-detector diffractometer
Absorption correction: multi-scan (*SADABS*; Bruker, 2002 ▶) *T*_min_ = 0.251, *T*_max_ = 0.347
13298 measured reflections
2673 independent reflections
2497 reflections with *I* > 2σ(*I*)
*R*_int_ = 0.048

#### Refinement

*R*[*F* ^2^ > 2σ(*F* ^2^)] = 0.030
*wR*(*F* ^2^) = 0.069
*S* = 1.02
2673 reflections
127 parameters
H-atom parameters constrained
Δρ_max_ = 1.31 e Å^−3^
Δρ_min_ = −0.72 e Å^−3^
Absolute structure: Flack (1983 ▶), 1118 Friedel pairs
Flack parameter: 0.00 (1)

### 

Data collection: *SMART* (Bruker, 2002 ▶); cell refinement: *SAINT* (Bruker, 2003 ▶); data reduction: *SAINT*; program(s) used to solve structure: *SHELXS97* (Sheldrick, 2008 ▶); program(s) used to refine structure: *SHELXL97* (Sheldrick, 2008 ▶); molecular graphics: *X-SEED* (Barbour, 2001 ▶); software used to prepare material for publication: *SHELXL97* and *PLATON* (Spek, 2003 ▶).

## Supplementary Material

Crystal structure: contains datablocks global, I. DOI: 10.1107/S1600536808012828/dn2343sup1.cif

Structure factors: contains datablocks I. DOI: 10.1107/S1600536808012828/dn2343Isup2.hkl

Additional supplementary materials:  crystallographic information; 3D view; checkCIF report

**Fig. 1.**
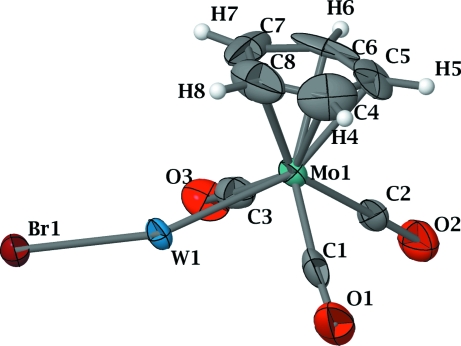
Molecular structure of the title complex showing the atom numbering scheme. Ellipsoids are drawn at the 50% probability level.

Enhanced figure: interactive version of Fig. 1

## supplementary crystallographic information

### Comment

The compound I was a by-product of a study on the functionalization of paraffins using transition metals. The functionalized compounds have potential applications in catalysis and organic syntheses (Changamu *et al.*, 2006). The compound I is similar to the reported structure of (η^5^-C_5_H_5_(CO)_3_MoHgCl (Bueno *et al.*, 1981), Albright *et al.* (1978). The bond distances of W—Mo, 2.6872 (7) Å and W—Br, 2.5591 (9) Å are comparable to Hg—Mo, 2.693 (30) Å and Hg—Cl, 2.437 (8) Å respectively. The slight difference between the bond lenghts involving the halides could be attributed to the difference in electronegativity and hence basicity between bromine and chlorine.The coordination around Mo is a pseudo-square pyramidal piano stool arrangement.(Fig. 1)

### Experimental

The compound I was prepared according to a reported procedure (Friedrich *et al.*, 2004) and crystals were grown by slow evaporation of a mixture of dichloromethane and hexane at 263 K.

### Refinement

Hydrogen atoms were treated as riding on their parent C atoms with C–H = 0.95 Å and *U*_iso_(H) = 1.2 *U*_eq_(C).

### Crystal data

**Table:**

[WMoBr(C_5_H_5_)(CO)_3_]	*Z* = 8
*M**_r_* = 508.82	*F*_000_ = 1824
Tetragonal, *P*42_1_*c*	*D*_x_ = 3.051 Mg m^−^^3^
Hall symbol: P -4 2n	Mo *K*α radiation λ = 0.71073 Å
*a* = 11.9375 (9) Å	Cell parameters from 2238 reflections
*b* = 11.9375 (9) Å	θ = 2.2–25.5º
*c* = 15.546 (2) Å	µ = 15.09 mm^−^^1^
α = 90º	*T* = 100 (2) K
β = 90º	Block, yellow
γ = 90º	0.11 × 0.10 × 0.07 mm
*V* = 2215.4 (4) Å^3^	

### Data collection

**Table:**

Bruker APEX CCD area-detector diffractometer	2673 independent reflections
Radiation source: fine-focus sealed tube	2497 reflections with *I* > 2σ(*I*)
Monochromator: graphite	*R*_int_ = 0.048
*T* = 100(2) K	θ_max_ = 28.3º
ω scans	θ_min_ = 2.2º
Absorption correction: multi-scan(SADABS; Bruker, 2002)	*h* = −15→14
*T*_min_ = 0.251, *T*_max_ = 0.347	*k* = −8→15
13298 measured reflections	*l* = −20→18

### Refinement

**Table:**

Refinement on *F*^2^	Hydrogen site location: inferred from neighbouring sites
Least-squares matrix: full	H-atom parameters constrained
*R*[*F*^2^ > 2σ(*F*^2^)] = 0.030	*w* = 1/[σ^2^(*F*_o_^2^) + (0.0238*P*)^2^] where *P* = (*F*_o_^2^ + 2*F*_c_^2^)/3
*wR*(*F*^2^) = 0.069	(Δ/σ)_max_ = 0.001
*S* = 1.02	Δρ_max_ = 1.31 e Å^−^^3^
2673 reflections	Δρ_min_ = −0.72 e Å^−^^3^
127 parameters	Extinction correction: none
Primary atom site location: structure-invariant direct methods	Absolute structure: Flack (1983), 1118 Friedel pairs
Secondary atom site location: difference Fourier map	Flack parameter: 0.00 (1)

### Special details

**Table:**

Geometry. All e.s.d.'s (except the e.s.d. in the dihedral angle between two l.s. planes) are estimated using the full covariance matrix. The cell e.s.d.'s are taken into account individually in the estimation of e.s.d.'s in distances, angles and torsion angles; correlations between e.s.d.'s in cell parameters are only used when they are defined by crystal symmetry. An approximate (isotropic) treatment of cell e.s.d.'s is used for estimating e.s.d.'s involving l.s. planes.
Refinement. Refinement of *F*^2^ against ALL reflections. The weighted *R*-factor *wR* and goodness of fit *S* are based on *F*^2^, conventional *R*-factors *R* are based on *F*, with *F* set to zero for negative *F*^2^. The threshold expression of *F*^2^ > σ(*F*^2^) is used only for calculating *R*-factors(gt) *etc*. and is not relevant to the choice of reflections for refinement. *R*-factors based on *F*^2^ are statistically about twice as large as those based on *F*, and *R*- factors based on ALL data will be even larger.

### Fractional atomic coordinates and isotropic or equivalent isotropic displacement parameters (Å^2^)

**Table:**

	*x*	*y*	*z*	*U*_iso_*/*U*_eq_	
W1	0.64476 (3)	0.39974 (3)	0.89630 (2)	0.01742 (9)	
Mo1	0.73518 (6)	0.20182 (6)	0.85204 (5)	0.01833 (16)	
Br1	0.62054 (7)	0.61253 (7)	0.90359 (5)	0.01974 (17)	
O1	0.5124 (6)	0.1402 (6)	0.9503 (5)	0.0480 (19)	
O2	0.6348 (5)	0.0161 (6)	0.7340 (4)	0.0402 (17)	
O3	0.6948 (5)	0.3305 (6)	0.6794 (4)	0.0330 (16)	
C1	0.5937 (8)	0.1680 (7)	0.9122 (6)	0.031 (2)	
C2	0.6724 (7)	0.0833 (7)	0.7766 (6)	0.026 (2)	
C3	0.7057 (7)	0.2868 (8)	0.7450 (6)	0.027 (2)	
C4	0.8467 (10)	0.1507 (11)	0.9698 (7)	0.047 (3)	
H4	0.8151	0.1245	1.0222	0.057*	
C5	0.8729 (8)	0.0831 (8)	0.9024 (7)	0.038 (2)	
H5	0.8622	0.0043	0.8996	0.046*	
C6	0.9203 (7)	0.1535 (11)	0.8354 (6)	0.044 (3)	
H6	0.9468	0.1308	0.7805	0.052*	
C7	0.9181 (9)	0.2684 (10)	0.8715 (9)	0.059 (4)	
H7	0.9431	0.3359	0.8455	0.071*	
C8	0.8694 (10)	0.2533 (11)	0.9549 (7)	0.052 (3)	
H8	0.8557	0.3126	0.9943	0.063*	

### Atomic displacement parameters (Å^2^)

**Table:**

	*U*^11^	*U*^22^	*U*^33^	*U*^12^	*U*^13^	*U*^23^
W1	0.01888 (17)	0.01447 (16)	0.01889 (15)	0.00532 (12)	0.00265 (14)	−0.00152 (14)
Mo1	0.0158 (3)	0.0170 (3)	0.0222 (3)	0.0033 (3)	0.0015 (3)	−0.0020 (3)
Br1	0.0214 (4)	0.0168 (4)	0.0209 (4)	0.0015 (3)	0.0032 (3)	0.0010 (3)
O1	0.036 (4)	0.032 (4)	0.076 (5)	0.006 (3)	0.029 (4)	0.011 (4)
O2	0.034 (4)	0.033 (4)	0.054 (4)	0.006 (3)	−0.008 (3)	−0.016 (3)
O3	0.035 (4)	0.045 (4)	0.019 (3)	0.007 (3)	−0.005 (3)	0.002 (3)
C1	0.032 (5)	0.015 (4)	0.045 (6)	0.009 (4)	0.004 (5)	0.003 (4)
C2	0.020 (5)	0.020 (5)	0.037 (5)	0.003 (4)	0.006 (4)	−0.012 (4)
C3	0.020 (5)	0.033 (5)	0.029 (5)	0.002 (4)	−0.004 (4)	−0.008 (4)
C4	0.042 (6)	0.069 (8)	0.030 (5)	−0.002 (7)	0.000 (5)	0.002 (6)
C5	0.033 (5)	0.030 (5)	0.053 (6)	0.015 (4)	−0.025 (5)	0.004 (5)
C6	0.016 (5)	0.091 (9)	0.024 (5)	0.028 (5)	−0.007 (4)	−0.007 (5)
C7	0.025 (6)	0.043 (7)	0.110 (11)	−0.014 (5)	−0.036 (6)	0.048 (7)
C8	0.037 (7)	0.058 (8)	0.061 (7)	0.015 (6)	−0.016 (5)	−0.035 (7)

### Geometric parameters (Å, °)

**Table:**

W1—Br1	2.5591 (9)	O3—C3	1.152 (12)
W1—Mo1	2.6872 (7)	C4—C8	1.276 (17)
Mo1—C1	1.972 (10)	C4—C5	1.359 (15)
Mo1—C3	1.980 (10)	C4—H4	0.9500
Mo1—C2	1.985 (8)	C5—C6	1.453 (15)
Mo1—C6	2.298 (8)	C5—H5	0.9500
Mo1—C5	2.307 (8)	C6—C7	1.482 (17)
Mo1—C7	2.344 (10)	C6—H6	0.9500
Mo1—C4	2.345 (12)	C7—C8	1.433 (16)
Mo1—C8	2.346 (10)	C7—H7	0.9500
O1—C1	1.184 (11)	C8—H8	0.9500
O2—C2	1.132 (10)		
			
Br1—W1—Mo1	158.35 (3)	C4—Mo1—W1	104.9 (3)
C1—Mo1—C3	110.5 (4)	C8—Mo1—W1	82.5 (3)
C1—Mo1—C2	79.1 (4)	O1—C1—Mo1	175.0 (8)
C3—Mo1—C2	78.5 (4)	O2—C2—Mo1	178.9 (8)
C1—Mo1—C6	145.6 (4)	O3—C3—Mo1	174.3 (8)
C3—Mo1—C6	101.8 (4)	C8—C4—C5	112.4 (11)
C2—Mo1—C6	96.8 (4)	C8—C4—Mo1	74.3 (7)
C1—Mo1—C5	108.9 (4)	C5—C4—Mo1	71.5 (6)
C3—Mo1—C5	136.6 (4)	C8—C4—H4	123.8
C2—Mo1—C5	91.8 (4)	C5—C4—H4	123.8
C6—Mo1—C5	36.8 (4)	Mo1—C4—H4	121.9
C1—Mo1—C7	143.7 (4)	C4—C5—C6	107.4 (10)
C3—Mo1—C7	95.8 (4)	C4—C5—Mo1	74.5 (6)
C2—Mo1—C7	132.1 (4)	C6—C5—Mo1	71.3 (5)
C6—Mo1—C7	37.2 (4)	C4—C5—H5	126.3
C5—Mo1—C7	60.0 (4)	C6—C5—H5	126.3
C1—Mo1—C4	93.6 (4)	Mo1—C5—H5	119.8
C3—Mo1—C4	152.9 (4)	C5—C6—C7	104.9 (9)
C2—Mo1—C4	119.4 (4)	C5—C6—Mo1	71.9 (5)
C6—Mo1—C4	58.5 (4)	C7—C6—Mo1	73.0 (5)
C5—Mo1—C4	34.0 (4)	C5—C6—H6	127.6
C7—Mo1—C4	57.2 (4)	C7—C6—H6	127.6
C1—Mo1—C8	108.4 (4)	Mo1—C6—H6	119.6
C3—Mo1—C8	124.1 (4)	C8—C7—C6	103.5 (9)
C2—Mo1—C8	148.0 (4)	C8—C7—Mo1	72.3 (6)
C6—Mo1—C8	59.1 (4)	C6—C7—Mo1	69.7 (5)
C5—Mo1—C8	56.1 (4)	C8—C7—H7	128.2
C7—Mo1—C8	35.6 (4)	C6—C7—H7	128.2
C4—Mo1—C8	31.6 (4)	Mo1—C7—H7	121.7
C1—Mo1—W1	73.4 (2)	C4—C8—C7	111.8 (10)
C3—Mo1—W1	72.1 (3)	C4—C8—Mo1	74.2 (7)
C2—Mo1—W1	128.8 (3)	C7—C8—Mo1	72.1 (6)
C6—Mo1—W1	129.5 (3)	C4—C8—H8	124.1
C5—Mo1—W1	137.7 (3)	C7—C8—H8	124.1
C7—Mo1—W1	92.5 (3)	Mo1—C8—H8	121.1
			
Br1—W1—Mo1—C1	−172.7 (3)	C8—Mo1—C6—C5	73.6 (7)
Br1—W1—Mo1—C3	−54.0 (3)	W1—Mo1—C6—C5	119.9 (6)
Br1—W1—Mo1—C2	−112.2 (3)	C1—Mo1—C6—C7	−115.9 (9)
Br1—W1—Mo1—C6	36.8 (3)	C3—Mo1—C6—C7	83.9 (7)
Br1—W1—Mo1—C5	87.2 (4)	C2—Mo1—C6—C7	163.6 (6)
Br1—W1—Mo1—C7	41.3 (3)	C5—Mo1—C6—C7	−112.4 (8)
Br1—W1—Mo1—C4	97.9 (3)	C4—Mo1—C6—C7	−76.0 (7)
Br1—W1—Mo1—C8	75.5 (3)	C8—Mo1—C6—C7	−38.9 (6)
C1—Mo1—C4—C8	−120.1 (8)	W1—Mo1—C6—C7	7.4 (7)
C3—Mo1—C4—C8	33.5 (13)	C5—C6—C7—C8	−0.4 (9)
C2—Mo1—C4—C8	160.4 (7)	Mo1—C6—C7—C8	65.0 (7)
C6—Mo1—C4—C8	81.2 (8)	C5—C6—C7—Mo1	−65.4 (6)
C5—Mo1—C4—C8	120.8 (11)	C1—Mo1—C7—C8	8.5 (10)
C7—Mo1—C4—C8	36.9 (7)	C3—Mo1—C7—C8	145.7 (7)
W1—Mo1—C4—C8	−46.3 (8)	C2—Mo1—C7—C8	−134.5 (8)
C1—Mo1—C4—C5	119.1 (7)	C6—Mo1—C7—C8	−112.3 (8)
C3—Mo1—C4—C5	−87.3 (11)	C5—Mo1—C7—C8	−72.6 (7)
C2—Mo1—C4—C5	39.6 (8)	C4—Mo1—C7—C8	−32.7 (6)
C6—Mo1—C4—C5	−39.6 (7)	W1—Mo1—C7—C8	73.4 (7)
C7—Mo1—C4—C5	−83.8 (8)	C1—Mo1—C7—C6	120.8 (8)
C8—Mo1—C4—C5	−120.8 (11)	C3—Mo1—C7—C6	−102.0 (6)
W1—Mo1—C4—C5	−167.1 (6)	C2—Mo1—C7—C6	−22.2 (8)
C8—C4—C5—C6	0.7 (13)	C5—Mo1—C7—C6	39.7 (6)
Mo1—C4—C5—C6	64.2 (6)	C4—Mo1—C7—C6	79.6 (7)
C8—C4—C5—Mo1	−63.4 (10)	C8—Mo1—C7—C6	112.3 (8)
C1—Mo1—C5—C4	−67.1 (8)	W1—Mo1—C7—C6	−174.3 (6)
C3—Mo1—C5—C4	138.6 (7)	C5—C4—C8—C7	−1.0 (14)
C2—Mo1—C5—C4	−146.2 (7)	Mo1—C4—C8—C7	−62.8 (8)
C6—Mo1—C5—C4	115.0 (10)	C5—C4—C8—Mo1	61.8 (9)
C7—Mo1—C5—C4	74.7 (8)	C6—C7—C8—C4	0.9 (12)
C8—Mo1—C5—C4	32.8 (7)	Mo1—C7—C8—C4	64.1 (9)
W1—Mo1—C5—C4	18.7 (9)	C6—C7—C8—Mo1	−63.2 (6)
C1—Mo1—C5—C6	177.9 (6)	C1—Mo1—C8—C4	65.5 (8)
C3—Mo1—C5—C6	23.6 (8)	C3—Mo1—C8—C4	−162.4 (7)
C2—Mo1—C5—C6	98.8 (6)	C2—Mo1—C8—C4	−33.5 (12)
C7—Mo1—C5—C6	−40.2 (6)	C6—Mo1—C8—C4	−79.1 (8)
C4—Mo1—C5—C6	−115.0 (10)	C5—Mo1—C8—C4	−35.3 (7)
C8—Mo1—C5—C6	−82.2 (7)	C7—Mo1—C8—C4	−119.8 (10)
W1—Mo1—C5—C6	−96.2 (7)	W1—Mo1—C8—C4	135.2 (7)
C4—C5—C6—C7	−0.1 (10)	C1—Mo1—C8—C7	−174.7 (7)
Mo1—C5—C6—C7	66.2 (6)	C3—Mo1—C8—C7	−42.6 (8)
C4—C5—C6—Mo1	−66.3 (7)	C2—Mo1—C8—C7	86.3 (11)
C1—Mo1—C6—C5	−3.5 (10)	C6—Mo1—C8—C7	40.7 (6)
C3—Mo1—C6—C5	−163.7 (6)	C5—Mo1—C8—C7	84.5 (7)
C2—Mo1—C6—C5	−84.0 (6)	C4—Mo1—C8—C7	119.8 (10)
C7—Mo1—C6—C5	112.4 (8)	W1—Mo1—C8—C7	−105.0 (7)
C4—Mo1—C6—C5	36.5 (6)		
